# Dysregulated skeletal muscle myosin super-relaxation and energetics in male participants with type 2 diabetes mellitus

**DOI:** 10.1007/s00125-025-06436-0

**Published:** 2025-04-28

**Authors:** Christopher T. A. Lewis, Roger Moreno-Justicia, Lola Savoure, Enrique Calvo, Agata Bak, Jenni Laitila, Robert A. E. Seaborne, Steen Larsen, Hiroyuki Iwamoto, Marina Cefis, Jose A. Morais, Gilles Gouspillou, Jorge Alegre-Cebollada, Thomas J. Hawke, Jesús Vazquez, Miquel Adrover, Vincent Marcangeli, Rami Hammad, Jordan Granet, Pierrette Gaudreau, Mylène Aubertin-Leheudre, Marc Bélanger, Richard Robitaille, Atul S. Deshmukh, Julien Ochala

**Affiliations:** 1 Department of Biomedical Sciences, University of Copenhagen, Copenhagen, Denmark; 2 Novo Nordisk Foundation Center for Basic Metabolic Research, Faculty of Health and Medical Sciences, University of Copenhagen, Copenhagen, Denmark; 3 Centro Nacional de Investigaciones Cardiovasculares (CNIC), Madrid, Spain; 4 Centro de Investigación Biomédica en Red de Enfermedades Cardiovasculares (CIBERCV), Madrid, Spain; 5 Centre for Human and Applied Physiological Sciences, Faculty of Life Sciences & Medicine, King’s College London, London, UK; 6 Clinical Research Centre, Medical University of Bialystok, Bialystok, Poland; 7 SPring-8, Japan Synchrotron Radiation Research Institute, Hyogo, Japan; 8 Département des Sciences de l’Activité Physique, Faculté des Sciences, L’Université du Québec à Montréal (UQAM), Montréal, PQ Canada; 9 Groupe de Recherche en Activité Physique Adaptée, Montréal, PQ Canada; 10 Department of Medicine, Research Institute of the McGill University Health Centre, Montréal, PQ Canada; 11 Department of Pathology and Molecular Medicine, McMaster University, Hamilton, ON Canada; 12 Institut Universitari d’Investigació en Ciències de la Salut (IUNICS), Institut d’Investigació Sanitària Illes Balears (IdISBa), Departament de Química, Universitat de les Illes Balears, Palma de Mallorca, Spain; 13 Département des Sciences Biologiques, Faculté des Sciences, L’Université du Québec à Montréal (UQAM), Montréal, PQ Canada; 14 Centre de Recherche de l’Institut Universitaire de Gériatrie de Montréal, Montréal, PQ Canada; 15 Al-Ahliyya Amman University, Faculty of Educational Sciences, Department of Physical and Health Education, Amman, Jordan; 16 Centre de Recherche du Centre Hospitalier de l’Université de Montréal, Département de médecine, Université de Montréal, Montréal, PQ Canada; 17 Département de Neurosciences, Université de Montréal, Montréal, PQ Canada; 18 Centre Interdisciplinaire de Recherche sur le Cerveau et l’Apprentissage, Université de Montréal, Montréal, PQ Canada

**Keywords:** Diabetes, Metabolism, Myosin, Skeletal muscle

## Abstract

**Aims/hypothesis:**

Disrupted energy balance is critical for the onset and development of type 2 diabetes mellitus. Understanding of the exact underlying metabolic mechanisms remains incomplete, but skeletal muscle is thought to play an important pathogenic role. As the super-relaxed state of its most abundant protein, myosin, regulates cellular energetics, we aimed to investigate whether it is altered in individuals with type 2 diabetes.

**Methods:**

We used vastus lateralis biopsy specimens (obtained from patients with type 2 diabetes and control participants with similar characteristics), and ran a combination of structural and functional assays consisting of loaded 2′- (or 3′)-*O-*(*N-*methylanthraniloyl)-ATP (Mant-ATP) chase experiments, x-ray diffraction and LC-MS/MS proteomics in isolated muscle fibres.

**Results:**

Our studies revealed a greater muscle myosin super-relaxation and decreased ATP demand in male participants with type 2 diabetes than in control participants. Subsequent proteomic analyses indicated that these (mal)adaptations probably originated from remodelled sarcomeric proteins and greater myosin glycation levels in patients than in control participants.

**Conclusions/interpretation:**

Overall, our findings indicate a complex molecular dysregulation of myosin super-relaxed state and energy consumption in male participants with type 2 diabetes. Ultimately, pharmacological targeting of myosin could benefit skeletal muscle and whole-body metabolic health through enhancement of ATP consumption.

**Data availability:**

The raw MS data have been deposited to the ProteomeXchange Consortium via the PRIDE partner repository with the dataset identifier PXD053022.

**Graphical Abstract:**

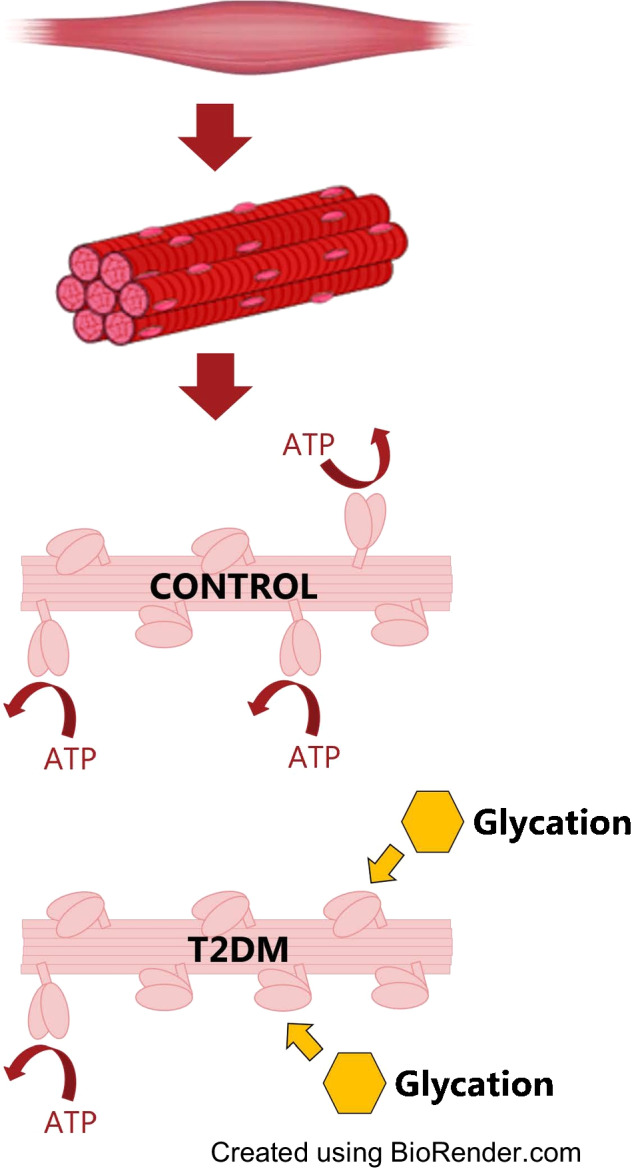

**Supplementary Information:**

The online version of this article (10.1007/s00125-025-06436-0) contains peer-reviewed but unedited supplementary material.


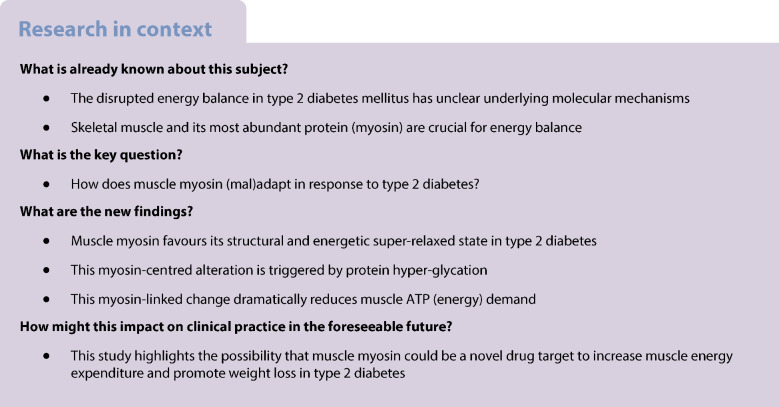


## Introduction

As a major tissue in the regulation of whole-body glycaemic control, skeletal muscle has long been established as critical to the onset of type 2 diabetes mellitus [1]. While decades of research have advanced our molecular understanding of how skeletal muscle contributes to the aetiology of such disease, few studies have investigated molecular changes in energy expenditure and whether the most abundant protein, myosin, is involved.

Skeletal muscle contraction is highly energy-dependent, and ATP is consumed directly by the head region of the myosin molecule, which is itself an ATPase [2]. Until recently, energy usage was thought to be mostly linked to the consumption of ATP by active myosin molecules. However, this doctrine has been challenged following the discovery that myosin maintains a significant consumption of ATP, and that the rate of this consumption is dependent on its resting state [3]. It is now established that myosin can adopt at least two different biochemical states at rest, known as the disordered-relaxed state (DRX) and the super-relaxed state (SRX) [3–5]. In the DRX, the myosin heads are likely to be in a structural ‘ON’ state. They are not bound to actin and exist freely in the interfilamentous space of the sarcomere [6, 7]. In the SRX, the head regions of the myosin molecules have a structural ‘OFF’ conformation in which they are folded backwards against the thick filament backbone of the sarcomere [6, 8]. This folding means that, in the SRX state, the ATPase site located on the head region of the myosin molecule is sterically inhibited, preventing ATP binding [7, 9]. Therefore, myosin molecules that are in the SRX have an ATP turnover rate that is approximately ten times slower than those in the DRX [4, 10, 11].

Muscle myosin molecules exist in a carefully controlled ratio of DRX to SRX [12]. Dysregulation of this ratio occurs in inherited conditions of both cardiac and skeletal muscle following mutations to genes encoding proteins of the sarcomere [13–15]. It has been estimated that a 20% shift of myosin heads from the SRX to the DRX in skeletal muscle would increase whole-body energy expenditure by 16% [4]. Thus, uncovering changes to the DRX:SRX ratio in metabolic diseases, such as type 2 diabetes, is of great interest, as it would improve understanding of major molecular disturbances in energy demand. Hence, in the present study, we aimed to explore the hypothesis that, in type 2 diabetes, there is a remodelling of the proportions of myosin molecules in the DRX and the SRX, ultimately affecting the ATP consumption of resting skeletal muscle.

The main cellular source of ATP is glycolysis, a process that is not harmless for cells, as it produces highly reactive carbonyl compounds as side products, including methylglyoxal (MG). MG has high intrinsic reactivity, and thus is one of the endogenous compounds with higher ability to randomly modify nucleophilic groups of long-life biomolecules [16]. Because of this, evolution has designed enzymatic mechanisms to control the levels of intracellular MG, such as the glyoxalase system (Glo-1 and Glo-2). However, both of these enzymes become downregulated in type 2 diabetes and, consequently, the levels of MG in diabetic individuals are two- to sixfold higher than in non-diabetic individuals [17]. Once it is formed, MG rapidly reacts with Arg, Lys and Cys side-chains, producing MG-derived advanced glycation end-products (AGEs), such as *N*^ε^-(carboxyethyl)lysine (CEL), methylglyoxal-derived hydroimidazolones (MG-Hs, which include MG-H1, MG-H2 and MG-H3) and 2-ammonio-6-[1-(5-ammonio-6-oxido-6-oxohexyl)-5-methylimidazolium-3-yl] hexanoate [18, 19]. Formation of these AGEs changes the chemical nature of the protein residues, potentially impacting the protein structure and thus their function, and thus potentially exacerbating the progression of type 2 diabetes [20]. Some studies have demonstrated that, in patients with type 2 diabetes, glycation on myosin can also occur, affecting its function [21, 22]. Therefore, we aimed to test the hypothesis that changes in the myosin DRX:SRX ratio in individuals with type 2 diabetes are intrinsically caused by aberrant levels of glycation.

## Methods

### Sample collection

Human vastus lateralis biopsies were collected from two cohorts. The collection of muscle biopsies from male control participants and male participants with type 2 diabetes in Denmark was approved by the ethical committee for the Capital Region of Denmark (H-15010122) and was conducted in accordance with the Declaration of Helsinki. All study participants were informed and signed a consent form before enrolment in the study. Characteristics of participants in this study and the biopsy procedure have been described previously [23, 24]. For collection of muscle biopsies from control participants and patients with type 2 diabetes in Canada, all procedures were conducted in accordance with the Declaration of Helsinki and approved by the Ethics Committee of the Université du Québec à Montréal (CIEREH-2020-3477). HOMA-IR was calculated as previously described [24]. Informed consent was obtained from all participants. Skeletal muscle biopsy samples were obtained from the vastus lateralis muscle under local anaesthesia using a suction modified Bergström needle performed under local anaesthesia. Samples were immediately frozen in liquid nitrogen and stored at −80°C until use. Sex was self-reported in both cohorts.

### Single muscle fibre preparation

Cryopreserved muscle samples were dissected into small sections and immersed in a membrane-permeabilising solution, described previously [15] (relaxing solution containing glycerol; 50:50 vol./vol.) for 24 h at −20°C, after which they were transferred to 4°C. The rigor buffer for 2′- (or 3′)-*O*-(*N*-methylanthraniloyl)-ATP (Mant-ATP) chase experiments contained 120 mmol/l potassium acetate, 5 mmol/l magnesium acetate, 2.5 mmol/l K_2_HPO_4_, 50 mmol/l MOPS, 2 mmol/l DTT at pH 6.8. These bundles were kept in the membrane-permeabilising solution at 4°C for another 24 h. After these steps, bundles were stored in the membrane-permeabilising buffer at −20°C for use within 2 weeks.

### Mant-ATP chase assay

Permeabilised skeletal muscle bundles were transferred to the relaxing solution and individual muscle fibres were isolated. Each muscle fibre was first incubated for 300 s with the rigor buffer described above. A solution containing the rigor buffer with added 250 μmol/l Mant-ATP was then added, and kept there for a further 300 s. This incubation time is chosen to ensure full incorporation of Mant-ATP into the sarcomere prior to the washout step. At the end of this step, another solution comprising the rigor buffer with 4 mmol/l ATP was added, with simultaneous acquisition of images.

For fluorescence acquisition, a Zeiss Axio Scope A1 microscope was used with a Plan-Apochromat 20×/0.8 objective and a Zeiss AxioCam ICm 1 camera. Frames were acquired every 5 s with a 20 ms acquisition/exposure time at 385 nm, for 300 s. These data were then fit to an unconstrained double exponential decay using GraphPad Prism version 9.0 (GraphPad.com):

$$\mathrm{normalised}\;\mathrm{fluorescence}\;=\;1\;-\;\mathrm P1\;\lbrack1-\exp(-t/\mathrm T1)\rbrack\;-\;\mathrm P2\;\lbrack1-\exp(-t/\mathrm T2)\rbrack$$

where P1 is the amplitude of the initial rapid decay, which approximates to the DRX, with T1 as the time constant for this decay, and P2 is the slower second decay approximating the proportion of myosin heads in the SRX, with the associated time constant T2. Mant-ATP chase experiments were performed at ambient laboratory temperature (20°C) for all samples.

**Fig. 1:**
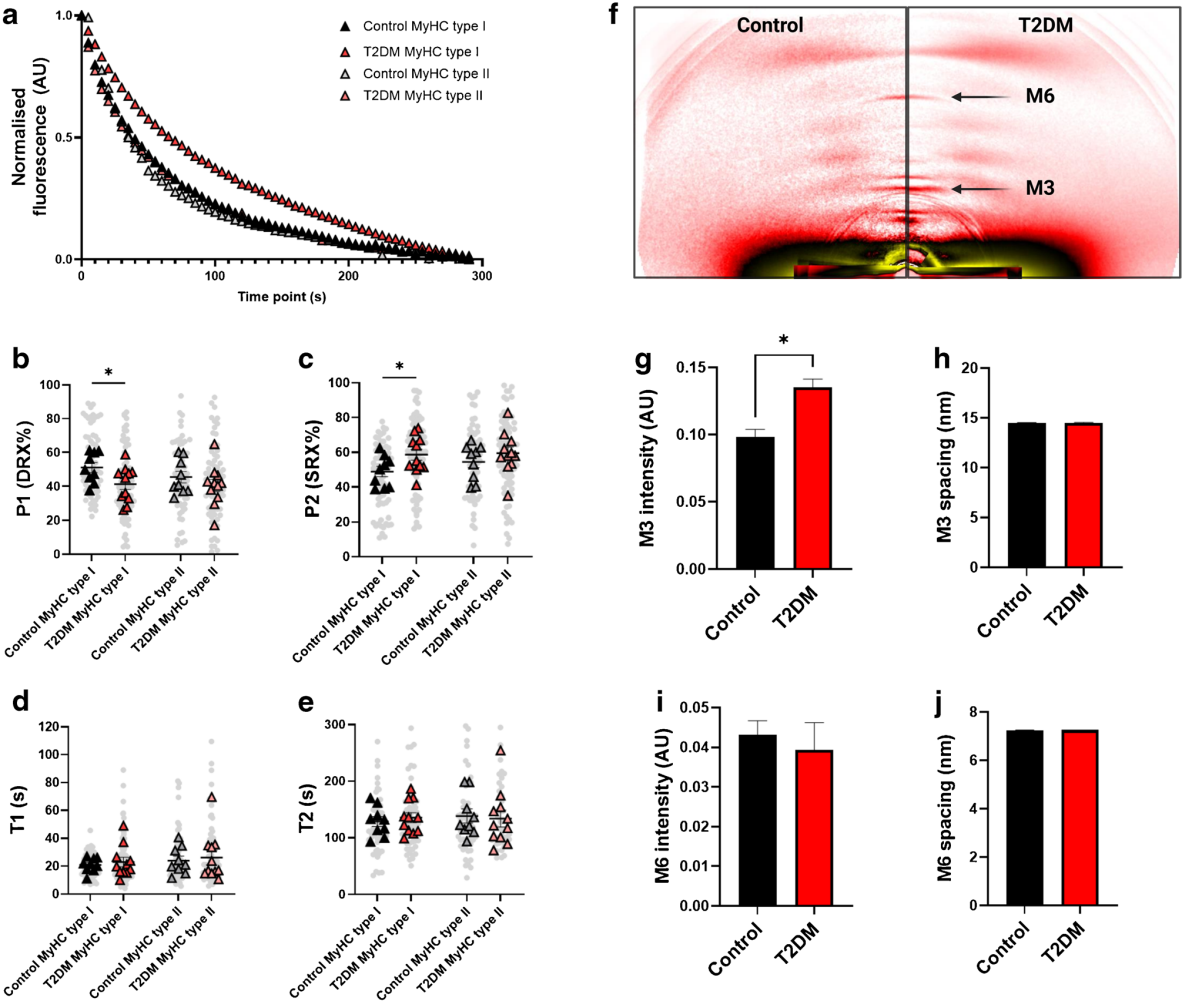
Myosin relaxed states are altered in type 2 diabetes (T2DM). (**a**) Representative fluorescence Mant-ATP decays from single muscle fibres isolated from skeletal muscle biopsies from control participants and participants with T2DM, measured over a 300 s period. (**b**, **c**) Percentage of myosin heads in P1/DRX (**b**) or P2/SRX (**c**) from single muscle fibres obtained from control participants and participants with T2DM. Values are separated based on fibre type: MyHC type I or MyHC type II. (**d**) T1 value indicating the ATP turnover lifetime for the DRX. (**e**) T2 value indicating the ATP turnover lifetime for the SRX. Grey circles represent the values from each individual muscle fibre analysed; coloured triangles represent the mean value for an individual participant; *n*=9–11 participants per group; 8–12 fibres were analysed per participant. An unpaired Student’s *t* test was used to calculate statistical significance of the differences among the mean values. (**f**) Representative x-ray diffraction recordings from permeabilised skeletal muscle bundles from control participants and those with T2DM. The M3 and M6 meridional reflections are indicated. (**g**) Normalised intensity (AU) of the M3 meridional reflection. (**h**) M3 meridional spacing, measured in nanometres (nm). (**i**) Normalised intensity (AU) of the M6 meridional reflection. (**j**) M6 meridional spacing, measured in nanometres (nm). Values are means ± SEM. All differences were tested for significance using a bilateral Student’s *t* test (*n*=6 participants per group): *p<0.05

Each isolated myofibre was then stained using an MYH7 antibody (A4.951, Developmental Studies Hybridoma Bank – DSHB; RRID: AB_528385) to determine whether the fibre was a type I or non-type I fibre, as previously described [25]. A representative image of positive staining in isolated single fibres is shown in electronic supplementary material (ESM) Fig. 1. Due to the relatively low abundance (<1%) of hybrid (type IIX) fibres in vastus lateralis human biopsies, we considered all non-type I fibres to be type II (fast) fibres [26]. Furthermore, our single-fibre proteomics data indicate the presence of zero pure type IIX fibres in this analysis.

### Synthesis and purification of MG

MG was purchased from Sigma-Aldrich as a solution at 40% in H_2_O (M0252). This solution was then further purified by steam distillation. The collected fractions were adjusted to pH 7.0 with NaOH, and aliquots of them were reacted with H_2_O_2_ in order to indirectly quantify the pure MG concentration by titration with KMnO_4_ [27].

### Acute glycation Mant-ATP chase assay

Fibres were incubated for 300 s in a modified relaxing buffer solution containing 5.89 mmol/l Na_2_ATP, 6.48 mmol/l MgCl_2_, 40.76 mmol/l propionic acid, 100 mmol/l *N*,*N*-bis(2-hydroxyethyl)-2-aminoethanesulfonic acid sodium salt, 6.97 mmol/l EGTA, 14.5 mmol/l sodium creatine phosphate dibasic and KOH to adjust the pH to 7.1 [28]. After this incubation, fibres then went through the same protocol as described above (incubation in rigor buffer and then Mant-ATP buffer, followed by fluorescence image acquisition). Then the same fibres were incubated in a modified relaxing buffer that contained 50 mmol/l MG for 30 min to induce acute glycation of the proteins in these muscle fibres. This concentration was selected based on previous literature that used MG synthesised via the same protocol [29]. This buffer contained 5.89 mmol/l Na_2_ATP, 6.48 mmol/l MgCl_2_, 40.76 mmol/l propionic acid, 100 mmol/l *N*,*N*-bis(2-hydroxyethyl)-2-aminoethanesulfonic acid sodium salt, 6.97 mmol/l EGTA, 43.5 mmol/l sodium chloride, 50 mmol/l MG and KOH to adjust the pH to 7.4.

Following this incubation, these muscle fibres were incubated for 300 s in the modified relaxing buffer described above, and then went through the same process of incubation in rigor buffer, incubation with Mant-ATP buffer, and then fluorescence image acquisition. For analysis purposes, the results for each individual muscle fibre were paired before and after MG incubation to observe the effects of acute glycation upon myosin dynamics.

### X-ray diffraction recordings and analysis

Thin muscle bundles were mounted into a specimen chamber in relaxing buffer, and then clamped at a sarcomere length of 2.00 μm. Subsequently, x-ray diffraction patterns were recorded at 15°C using a CMOS camera (model C11440-22CU, Hamamatsu Photonics, Japan; 2048 × 2048 pixels) in combination with a 4-inch image intensifier (model V7739PMOD, Hamamatsu Photonics). The x-ray wavelength was 0.10 nm, and the specimen-to-detector distance was 2.14 m. For each preparation, approximately 20–50 diffraction patterns were recorded at the BL40XU beamline of SPring-8 (Japan Synchrotron Radiation Research Institute, and were analysed as described previously [30]. To minimise radiation damage, the exposure time was kept low (0.5 or 1 s), and the specimen chamber was moved by 100 μm after each exposure. After x-ray recordings, background scattering was subtracted, and the major myosin meridional reflection intensities/spacing were determined as described previously [30].

### Single-fibre proteomics

Proteomics measurements were conducted using a previously described workflow [31]. In brief, an Evosep One HPLC system (Evosep), coupled via electrospray ionisation to a timsTOF SCP mass spectrometer (Bruker), was used as the LC-MS system. Peptides were separated using the ‘60 samples per day’ chromatographic method [31] before electrospray ionisation using a CaptiveSpray ion source and a 10 μm emitter directly within the MS instrument. Samples were then measured using a dia-PASEF workflow described elsewhere [31]. Processing of the raw MS spectra was performed using DIA-NN (version 1.8) in library-based mode, based on an in-house muscle fibre-specific MS library comprising 5000 proteins [32]. The DIA-NN settings were as follows: double-pass mode was selected as the neural network mode, ‘robust LC (high accuracy)’ was chosen as the quantification strategy, prototypic peptides were selected for quantification, the ‘match between runs’ option was enabled and the precursor FDR control was set to 1%. Unless specified, the other parameters remained as default settings.

### Single-fibre proteomics data analysis/statistical testing

Data analysis was performed using R software (version 4.3.2) and multiple packages from the R environment. The protein groups matrix was loaded into the software, and proteins were log_2_-transformed before being filtered for at least 70% valid values in at least one of the tested groups (type 2 diabetes or control participants). Next, the filtered protein matrix followed the limma (version 3.54.2) workflow, which included quantile normalisation of all samples and differential expression testing between groups in a pseudobulk manner [33]. To adjust *p* values for multiple comparison testing, we applied the Benjamini–Hochberg correction for the type I–type II comparison, and the Xiao significance score for the fibre type-specific comparisons of control participants vs those with type 2 diabetes, which takes into consideration both expression fold change and statistical significance. Proteins with a Xiao score under 0.05 were considered as differentially expressed between groups [34].

### Myosin heavy chain band isolation for post-translational identifications

Muscle biopsy samples were cut into 15 mg sections, and immersed in a sample buffer (0.5 M Tris buffer at pH 6.8, 0.5 mg/ml bromophenol blue, 10% SDS, 10% glycerol, 1.25% mercaptoethanol) at 4°C. Samples were then homogenised and centrifuged (1200 *g*), allowing the supernatant to be extracted and used for SDS–PAGE gels using a stacking gel made with acrylamide/bis-acrylamide 37.5:1 and separation gel made with acrylamide/bis-acrylamide 100:1). Proteins were separated, and the individual MYH7 band was excised manually [35].

### MS-based glycation peptide mapping

SDS–PAGE bands corresponding to MYH7 from the control or diabetic groups were subjected to in-gel digestion. After reduction with DTT (10 mmol/l) and alkylation of Cys groups with iodoacetamide (50 mmol/l), modified porcine trypsin (Promega) was added at a final ratio of 1:20 (trypsin:protein). Digestion proceeded overnight at 37°C in 100 mmol/l ammonium bicarbonate at pH 7.8. The resulting tryptic peptides were loaded and washed using Evotips, and separated on an Endurance EV1106 column (15 cm × 150 µm internal diameter; 1.9 µm beads) using an Evosep One HPLC system coupled to an Orbitrap Eclipse Tribrid mass spectrometer (Thermo Fisher) and a 30 SPD preprogrammed gradient.

MS analysis was performed using the data-independent scanning method as described previously [36], with some modifications. Each sample was analysed in a single chromatographic run covering a mass range from 390 to 1000 *m*/*z*. The cycle consisted of 255 sequential HCD MS/MS fragmentation events with 2.5 *m*/*z* windows from 390–900 *m*/*z*, and with 4 *m*/*z* windows from 900–1000 *m*/*z*. HCD fragmentation was performed using a 33 normalised collision energy. MS/MS scans were performed using a 70 ms injection time, a AGC target setting of 3 × 10^5^ ions and 17,500 resolutions. The whole cycle lasted a maximum of 18 s, depending on the ion intensity during chromatography. The narrow windows used for fragmentation allowed peptide identification using conventional DDA searching algorithms. The SEQUEST HT search program was used, as implemented in Proteome Discoverer 2.5 (Thermo Scientific), against a database containing human myosins and using the following parameters: two maximum missed trypsin cleavage sites, a precursor mass tolerance of 3 Da, and a fragment mass tolerance of 30 ppm. Oxidation on Met (+15.995 Da), CEL (+72.021 Da) and carboxymethyl (+58.005 Da) on Lys, and MG-Hs (+54.011 Da) on Arg were set as variable modifications. Carbamidomethylation (+57.021 Da) on Cys was set as a fixed modification [37].

### Statistical analysis

Data are presented as means ± SD. Graphs were prepared and analysed using GraphPad Prism version 9.0. Statistical significance was set to *p*<0.05 unless otherwise stated. Where data are unevenly distributed, a Mann–Whitney test was applied to assess differences between two groups. Where data are evenly distributed, a bilateral Student’s *t* test was used to assess differences between two groups; a paired Student’s *t* test was used in paired experiments. For analyses involving multiple groups, two-way ANOVAs with Šídák’s multiple comparisons test were used. The statistical test applied in each analysis is listed in each figure legend. The number of samples used in each experiment is listed in each individual figure legend, and each sample refers to experimental work on tissue from separate individual participants. Where replicates have been performed upon the same tissue from one individual participant, this is also stated in the figure legend. Throughout the study, experiments were performed unblinded.

## Results

Our experiments investigated individual skeletal muscle fibres from the vastus lateralis of male participants with type 2 diabetes and control participants, who were of a similar age and had similar BMI (Table 1). We focused on only one sex to limit the complexity of the study.

**Table 1:** Characteristics of male participants with type 2 diabetes and male control participants from whom muscle biopsy samples were obtained

	Control participants	T2DM patients
Age (years)	67.9 ± 12.9	67.9 ± 11.4
BMI (kg/m^2^)	29.76 ± 2.7	29.91 ± 2.4
Fat (%)	34.4 ± 3.7	33.1 ± 6.2
Muscle mass (kg)	57.89 ± 7.3	58.53 ± 7.7
HbA_1c_ (mmol/mol)	39.5 ± 4.0	54.4 ± 5.2***
HbA_1c_ (%)	5.9 ± 0.3	7.3 ± 0.5***
HOMA-IR	1.64 ± 0.9	2.58 ± 0.7
Glucose (mmol/l)	3.36 ± 3.0	6.4 ± 0.3**
Insulin (pmol/l)	35.12 ± 19.7	54.8 ± 15.4
Total cholesterol (mmol/l)	2.01 ± 1.7	3.67 ± 0.3
HDL-cholesterol (mmol/l)	0.79 ± 0.6	1.36 ± 0.2
LDL-cholesterol (mmol/l)	1.04 ± 0.6	1.64 ± 0.4
Triacylglycerols (mmol/l)	0.95 ± 0.5	1.44 ± 0.5
MyHC type I (%)	50.21 ± 10.03	43.93 ± 13.16*
MyHC type II (%)	49.79 ± 10.03	56.07 ± 13.16*

Values are means ± SD. A bilateral Student’s *t* test was used to calculate statistical significance: *p<0.05, **p<0.01, ***p<0.001. *n*=9–11 participants per group

Note that patients with type 2 diabetes (T2DM) had significantly higher HbA_1c_ and blood glucose than control participants. However, as these patients were being treated for T2DM, certain metrics did not exhibit differences (e.g. HOMA-IR)

### Participants with type 2 diabetes have higher levels of myosin SRX/OFF state in type I muscle fibres

To test our hypothesis that the myosin DRX to SRX ratio may be altered in individuals with type 2 diabetes, we performed loaded Mant-ATP chase assays on isolated and permeabilised single muscle fibres (Fig. 1a). A total of 284 individual myofibres were tested (between 8 and 12 fibres per individual). A fibre type breakdown from these samples is shown in Table 1 and a typical fibre is shown in ESM Fig. 1. Interestingly, we found a significantly lower number of myosin heads in the DRX (P1) in type I fibres of participants with type 2 diabetes than in control participants (Fig. 1b). This finding was matched by a significantly higher percentage of fibres in the SRX (P2) in type I fibres of participants with type 2 diabetes than in control participants (Fig. 1c) (*p*=0.0387). Despite this, we did not observe any differences between the groups for the ATP turnover times of the myosin molecule in the DRX (T1) (Fig. 1d) or the SRX (T2) (Fig. 1e). These results demonstrate that, in type 2 diabetes, the resting myosin state undergoes remodelling, causing a decrease in the ATP consumption of type I (oxidative) fibres. Interestingly, when assessing potential correlations between myosin states and the characteristics listed in Table 1, we observed a significant positive linear correlation between the DRX (P1) and lean mass in control muscle fibres expressing the myosin heavy chain (MyHC) type I isoform only (ESM Fig. 2). No significant correlation was found for type II fibres or in participants with type 2 diabetes (ESM Fig. 2).

To obtain further structural insights and relate the above DRX/SRX observations to myosin ON/OFF states, we performed small-angle x-ray diffraction on the same muscle biopsy specimens and focused on myosin meridional reflections, namely M3 and M6 (Fig. 1f). We found that the M3 intensity was significantly greater in individuals with type 2 diabetes than in control participants (*p*=0.0118) (Fig. 1g, h). This finding suggests that, even though the distance between myosin crowns is preserved, myosin heads are more ordered along the thick filament (OFF state) [38]. We did not observe any change in M6 intensity or spacing between the groups (Fig. 1i, j), indicating maintenance of thick filament compliance/extensibility [38]. Altogether, our findings support the hypothesis that, in type I fibres of individuals with type 2 diabetes, myosin molecules adopt a preferred ATP-conserving SRX and OFF state without any other major thick filament disturbances.

### The coiled-coil domain of the MYH7 protein is glycated in individuals with type 2 diabetes

**Fig. 2:**
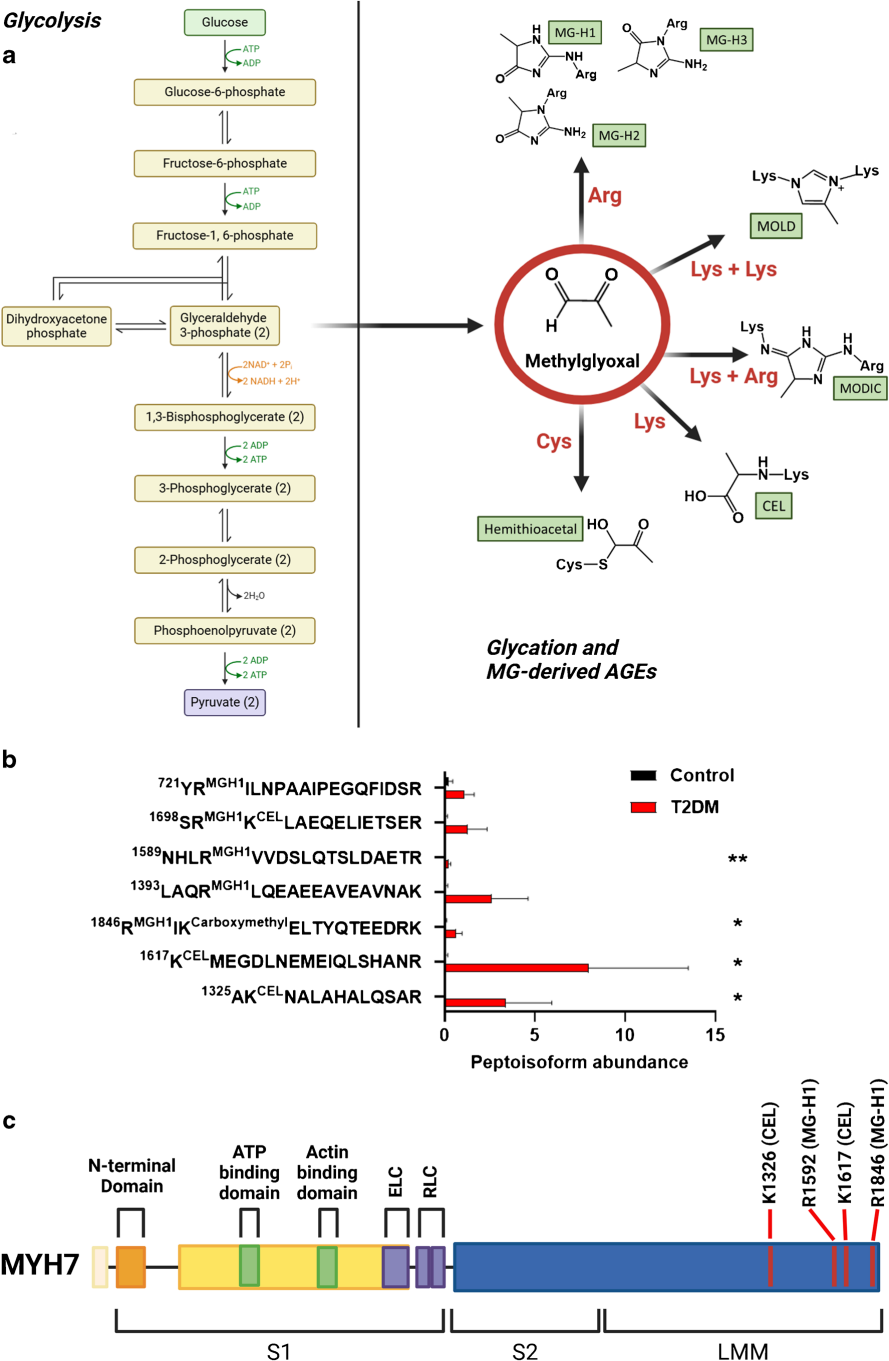
Glycation of type I myosin heavy chain (MYH7) in type 2 diabetes (T2DM). (**a**) Schematic of the process of glycolysis from which the intracellular MG is formed, and the chemical formulae of some of the MG-derived AGEs that have been detected on various protein residues. The figure was created using BioRender. (**b**) Quantification of several glycated peptides from MYH7, showing increased glycation in participants with T2DM when compared with control participants. The Mann–Whitney *U* test was used to calculate the statistical significance of the differences between groups: *p<0.05, **p<0.01 (*n*=5 participants per group). We detected several peptide fragments modified through formation of CEL, MG-Hs or *N*^ε^-(carboxymethyl)lysine. (**c**) Scheme of MYH7 protein regions, with the location of the glycated residues indicated in red. ELC, essential light chain; MOLD, methylglyoxal-lysine dimer; MODIC, (2-ammonio-6-([2-[(4-ammonio-5-oxido-5-oxopentyl)amino]-4-methyl-4,5-dihydro-1H-imidazol-5-ylidene]amino)hexanoate. RLC, regulatory light chain. The figure was created using BioRender

To get a deeper understanding of the molecular events leading to the changes in myosin resting states, we assessed the level of glycation on myosin in these samples. The schematic in Fig. 2a illustrates the formation of MG and MG-derived AGEs. We identified several AGE-modified peptides arising from myosin heavy chain type I (MYH7) protein obtained from patients with type 2 diabetes (ESM Fig. 3) that were absent in the control participants (Fig. 2b). These comprised CEL modifications on Lys residues K1326 and K1617 (*p*=0.0476 and *p*=0.0159, respectively) and MG-H1 modifications on Arg residues R1592 and R1846 (*p*=0.0317 and *p*=0.0079, respectively). The identification of these glycated peptides was in-depth validated by a highly sensitive scan using the VseqExplorer application to calculate quantitative E-scores. These glycated peptide fragments were mainly located in the coiled-coil region of the MYH7 protein (Fig. 2b, c).

### Acute glycation of type I muscle fibres induces an increase in the percentage of resting myosin heads in the SRX

**Fig. 3:**
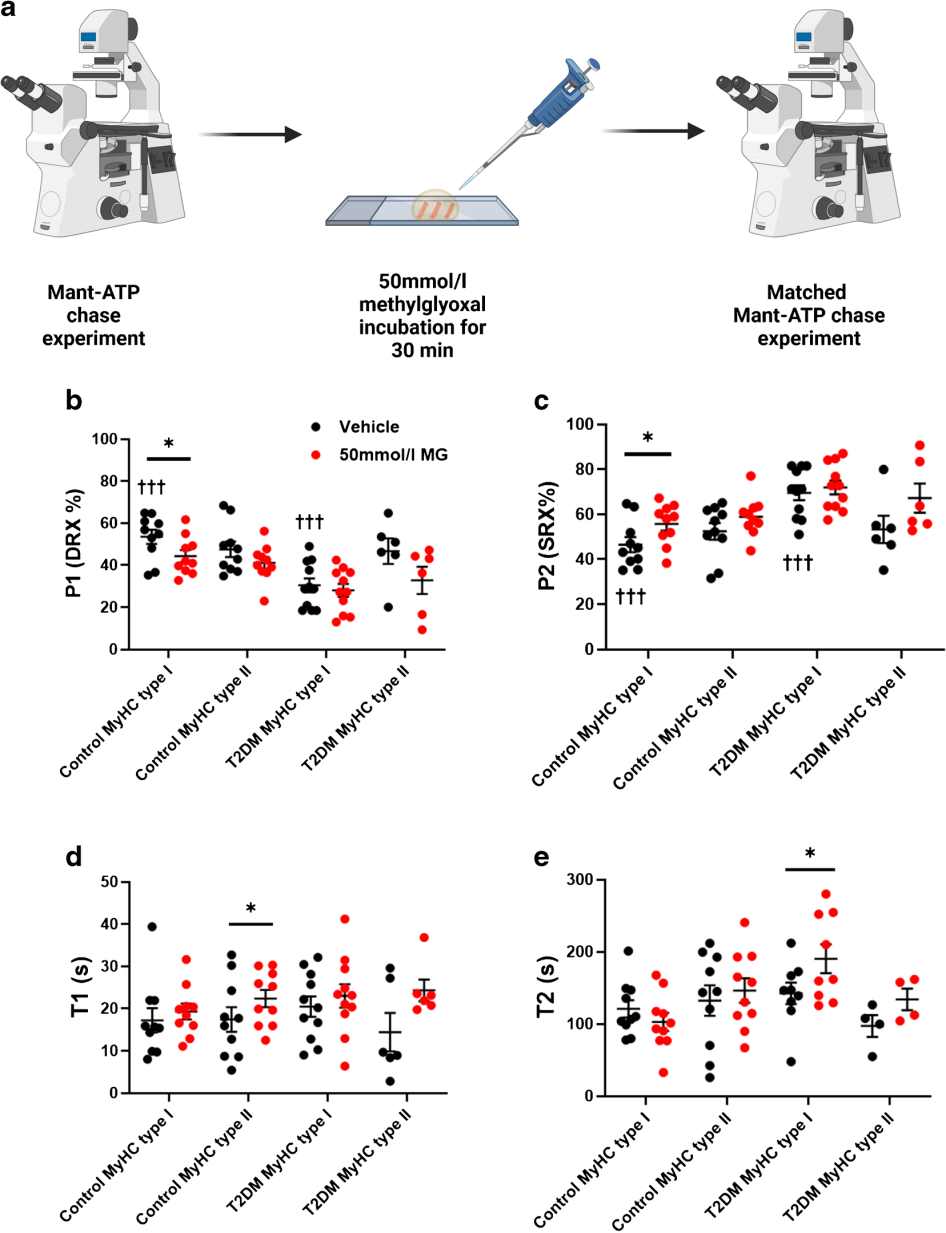
Acute glycation increased the percentage of myosin heads in the SRX in control participants. (**a**) Workflow for loaded Mant-ATP chase experiments in which single muscle fibres were incubated with 50 mmol/l MG in between matched experiments. The figure was created using BioRender. (**b**) Percentage of myosin heads in P1/DRX before (black circles) and after (red circles) treatment with MG. (**c**) Percentage of myosin heads in P2/SRX before (black circles) and after (red circles) treatment with MG. (**d**) T1 value indicating the ATP turnover lifetime of the DRX before (black circles) and after (red circles) treatment with MG. (**e**) T2 value indicating the ATP turnover lifetime of the SRX before (black circles) and after (red circles) treatment with MG. Values were separated based on fibre type: MyHC type I or MyHC type II. A paired Student’s *t* test was used to calculate statistical significance between fibres before and after MG incubation: *p<0.05. Two-way ANOVA with Šídák’s multiple comparisons test was used to calculate the significance between groups (between control and T2DM and between type I and type II fibres): ^†††^*p*<0.001 (*n*=4 participants per group)

To emphasise the functional effects of glycation on myosin proteins, we performed paired loaded Mant-ATP chase experiments after a 30 min incubation with and without MG (Fig. 3a). Consistent with our previous results, the baseline percentage of myosin heads in the DRX was lower in type I muscle fibres of participants with type 2 diabetes than in control participants (Fig. 3b). This was matched by a greater proportion of SRX in type I muscle fibres of participants with type 2 diabetes than in control participants (*p*<0.001) (Fig. 3c). Following incubation with MG, the fraction of myosin heads in the DRX in type I fibres from control participants significantly decreased but not that in type II fibres (Fig. 3b). Again, this was matched by a significant increase in the proportion of the SRX in type I fibres of control participants but not in type II fibres (*p*=0.0302) (Fig. 3c). However, we did not find any change following MG incubation for either fibre type in individuals with type 2 diabetes (Fig. 3b, c). A plausible reason for such discrepancy is that fibres from individuals with type 2 diabetes were already saturated and hyper-glycated at the time of the baseline experiments. MG incubations also had an impact on the ATP turnover time of the resting myosin molecules (Fig. 3d, e) (*p*=0.0123 and *p*=0.0190, respectively).

### Differential expression of sarcomeric proteins in type I muscle fibres of individuals with type 2 diabetes

**Fig. 4:**
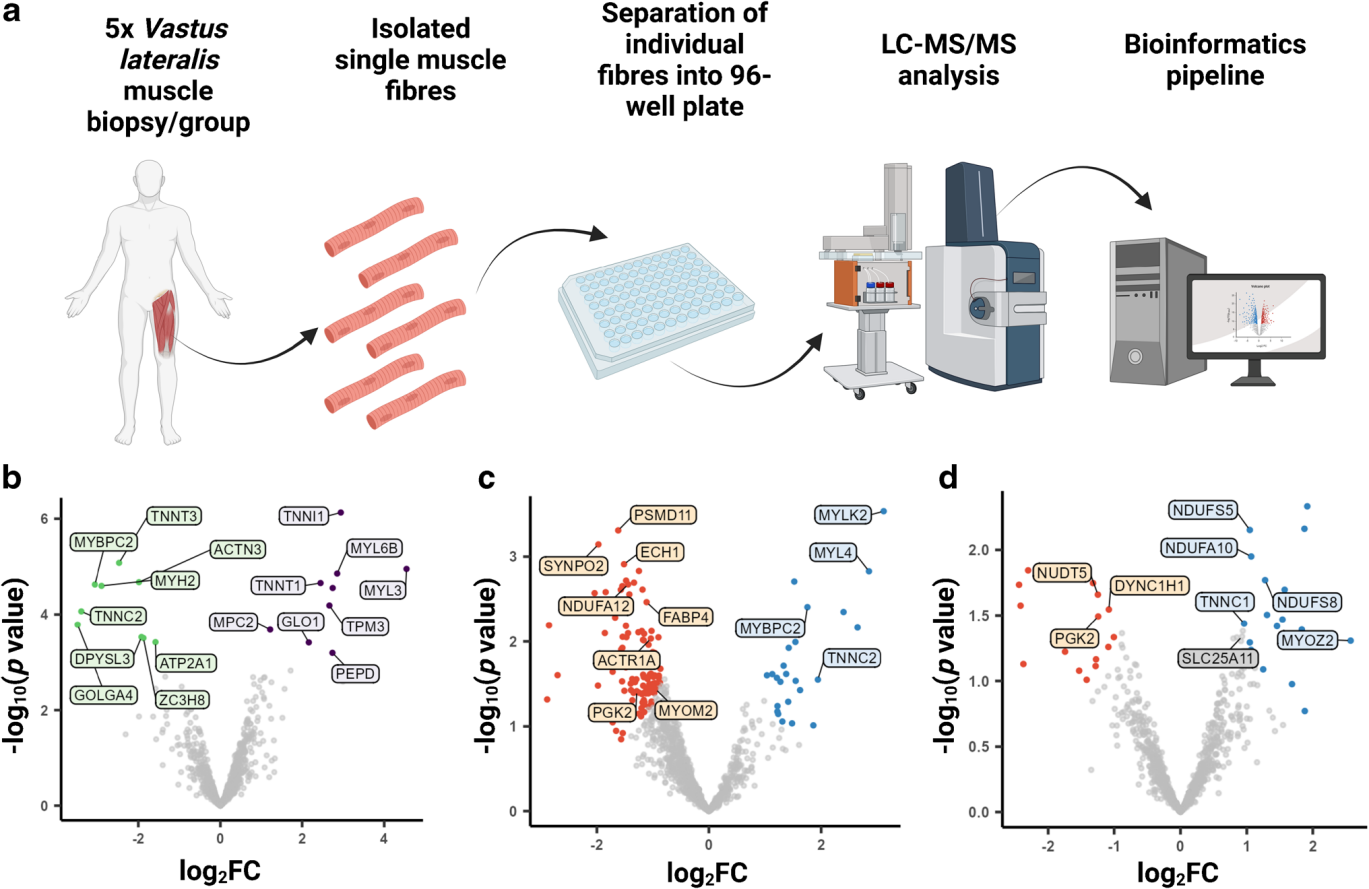
Single-fibre proteomics shows that type I muscle fibres from participants with type 2 diabetes (T2DM) have differential expression of sarcomeric proteins. (**a**) Workflow for isolation of single skeletal muscle fibres from vastus lateralis muscle biopsies, and downstream processing of these single muscle fibres using MS and a subsequent bioinformatics pipeline. The figure was created using BioRender. (**b**) Volcano plot showing differentially expressed proteins between type I and type II muscle fibres from both participant groups. (**c**) Volcano plot showing proteins that are differentially expressed in type I fibres between control and T2DM groups. (**d**) Volcano plot showing proteins that are differentially expressed in type II fibres between control and T2DM groups. Detailed information on statistical analysis of single-fibre proteomics is provided in Methods (*n*=5 participants per group). FC, fold change. ACTN3, α-actinin 3; ACTR1A, actin-related protein 1A; ATP2A1, sarcoplasmic/endoplasmic reticulum calcium ATPase 1; DPYSL3, dihydropyrimidinase-like 3; DYNC1H1, dynein cytoplasmic 1 heavy chain 1; ECH1, enoyl-CoA hydratase 1; FABP4, fatty acid binding protein 4; GLO1, glyoxalase I; GOLGA4, golgin subfamily A member 4; MPC2, mitochondrial pyruvate carrier 2; MYBPC2, myosin binding protein C2; MYH2, myosin 2; MYL3, myosin light chain 3; MYL4, myosin light chain 4; MYL6B, myosin light chain 6B; MYLK2, myosin light chain kinase 2; MYOM2, myomesin 2; MYOZ2, myozenin-2; NDUFA10, NADH:ubiquinone oxidoreductase subunit A10; NDUFA12, NADH:ubiquinone oxidoreductase subunit A12; NDUFS5, NADH:ubiquinone oxidoreductase subunit S5; NDUFS8, NADH:ubiquinone oxidoreductase core subunit S8; NUDT5, nudix hydrolase 5; PEPD, peptidase D; PGK2, phosphoglycerate kinase 2; PSMD11, 26S proteasome non-ATPase regulatory subunit 11; SLC25 A11, solute carrier family 25 member 11; SYNPO2, synaptopodin 2; TNNC1, troponin C1; TNNC2, troponin C2; TNNI1, troponin I1; TNNT1, troponin T1; TNNT3, troponin T3; TPM3, tropomyosin 3; ZC3H8, zinc finger CCCH-type containing 8

In addition to post-translational modifications and glycation, other more profound remodelling may occur in type 2 diabetes, and may include alterations in skeletal muscle protein expression. To investigate this, we performed high-throughput single muscle fibre proteomics using the same muscle biopsy specimens (Fig. 4a). A total of 109 single muscle fibres were analysed from ten different muscle biopsies (five per group). On average, 584 proteins were quantified in each single muscle fibre (ESM Table 1). The samples were grouped by fibre type as determined by immunohistochemistry, which was validated by the proteomics data. The validation revealed expected enrichment of known type I or II fibre-specific proteins, such as troponin T1 (TNNT1) and troponin I1 (TNNI1) or myosin 2 (MYH2) and myosin binding protein C2 (MYBPC2), respectively (Fig. 4b). This validation of the method via examination of expression profiles between fibre types provided a high level of confidence to proceed and look at the specific changes between the control and type 2 diabetes groups for each individual fibre type.

Interestingly, in type I muscle fibres, we identified 136 differentially expressed proteins between control participants and those with type 2 diabetes. Importantly, a large number of sarcomeric proteins (such as myosin light chain kinase 2 [MYLK2], MYBPC2 and myosin light chain 4 [MYL4]) were downregulated in type 2 diabetes muscle fibres (Fig. 4c, ESM Table 1). On the other hand, in type II myofibres, we observed 32 differentially expressed proteins between control participants and the type 2 diabetes group. Among these, mitochondrial-associated proteins such as the NADH:ubiquinone oxidoreductase subunit proteins NDUFS5, NDUFA10 and NDUFS8 were downregulated in type 2 diabetes (Fig. 4d, ESM Table 1). In summary, our analysis revealed distinct patterns of protein expression changes in type I and type II muscle fibres between control participants and those with type 2 diabetes.

## Discussion

In the present study, we aimed to identify whether resting myosin biochemical and structural states were altered in type 2 diabetes. We observed a higher proportion of myosin heads in the SRX and OFF state in patients with type 2 diabetes than in control participants. Interestingly, these alterations were only present in type I (slow oxidative) muscle fibres. The exact mechanisms underlying the increase in myosin super-relaxation may be complex, but are likely to involve type 2 diabetes-specific hyper-glycated residues on the coiled-coil region of MYH7. Indeed, when acute hyper-glycation was induced in isolated single muscle fibres, the percentage of myosin heads in the SRX increased. In addition to hyper-glycation, myosin super-relaxation in type 2 diabetes may also involve a remodelling of sarcomeric proteins as revealed by our single-fibre proteomics analyses.

The discovery of the myosin SRX has remarkably shifted the field of muscle cell biology, and implies a potential direct link between resting myosin conformation and the metabolic rate of skeletal muscle [3, 4]. However, much of the early work on the biophysics of resting myosin head states has been in cardiac diseases, particularly inherited hypertrophic cardiomyopathy [13, 14, 39]. It is only recently that we have demonstrated that myosin biochemical states are dynamic and greatly influenced by non-genetic factors. Based on these findings, investigating the potential involvement of changes to the dynamics of myosin in cardiometabolic syndromes such as type 2 diabetes was an obvious next step. Our primary finding, a higher fraction of myosin molecules in the SRX and OFF state in type 2 diabetes, is striking. Critically, this means that the type I fibres of individuals with type 2 diabetes have a reduced ATP turnover and thus energy consumption per muscle fibre. As skeletal muscle makes up around 40% of whole-body mass, even small changes in the energy consumption of this tissue can have significant consequences for whole-body energy expenditure [40, 41]. This is particularly interesting as patients with type 2 diabetes have been shown to lose weight to a lesser extent than overweight non-diabetic control participants, even when placed on matched weight management interventions [42]. Future studies in this field should focus on whether the resting myosin SRX/OFF state is altered in obesity without the background of type 2 diabetes, and whether dynamic changes to myosin conformation occur after weight loss and/or gain. Given these findings, it is tempting to suggest that restoring the SRX in the context of cardiometabolic syndromes would promote ATP consumption in muscle, and may favour a greater whole-body energy expenditure. This would then position myosin as a promising target for treating type 2 diabetes.

Despite its relatively recent discovery, there has already been clinical success in targeting the SRX, including the use of mavacamten, which is clinically approved for the treatment of hypertrophic cardiomyopathy [43, 44]. Novel pharmacological compounds that can directly increase the DRX, specifically in skeletal muscle, may therefore hold therapeutic potential for further conditions, such as type 2 diabetes. Interestingly, the molecule piperine, an alkaloid component of black pepper, has been shown to be able to bind the myosin regulatory light chain specifically in fast-twitch skeletal muscle to de-stabilise the SRX, and thus increase the proportion of myosin heads in the DRX [45–47]. Although piperine itself may not hold the chemical properties necessary to make it suitable for therapeutic intervention, the demonstration that a small molecule can achieve this mode of action is promising. Critically, in-depth in vitro studies that assess whether there is a direct link between changes to resting myosin conformation and molecular mechanisms that are impaired in the skeletal muscle of individuals with type 2 diabetes, such as insulin resistance, are required in order to confirm that such a modality would effectively treat type 2 diabetes. Such experiments could make use of the aforementioned compounds to pharmacologically alter the resting skeletal muscle myosin conformation in vitro.

Interestingly, the higher myosin super-relaxation only occurred in type I muscle fibres. Type I and type II fibres differ metabolically, with type I fibres having a higher density of mitochondria and thus favouring oxidative respiration, while type II fibres have a relatively higher glycolytic capacity [48]. Importantly, type I muscle fibres have a higher expression of GLUT4 and a far higher glucose-handling capacity compared with type II fibres [49]. Given that glucose handling by skeletal muscle is one of the critical processes that is be dysregulated during the development of type 2 diabetes, we believe that delineating whether myosin conformation and glucose handling in type 2 diabetes are biologically linked is essential to pursue in future studies.

The molecular mechanisms underlying the changes in resting myosin conformations may be complex, but our study highlights the potential role of post-translational modifications such as glycation. Previous studies by Papadaki et al showed that, in patients with type 2 diabetes, proteins of the cardiac myofilament were hyper-glycated, and that this hyper-glycation reduced cardiac contractility [21, 50]. In the SRX/OFF state, the myosin head is folded backwards, and is thus able to form interactions with the tail region [8, 39]. The functional and experimental relevance of these unusual post-translational modifications was demonstrated using MG [51]. When skeletal muscle fibres were treated with MG, we only observed an increased amount of myosin heads in the SRX state for type I muscle fibres of control participants (not for those with type 2 diabetes). It is highly plausible that, as a non-reversible covalent attachment of a carbohydrate or an α-oxo-aldehyde to an amino acid residue, the MG-treated myofibres from individuals with type 2 diabetes were already glycated/saturated prior to the incubation and thus were not significantly affected.

The proteomics analysis displayed differences in the expression of major sarcomeric proteins in type I skeletal muscle fibres of participants with type 2 diabetes compared with control participants. Downregulated proteins included MYLK2 and MYL4. MYLK2 is a kinase that, in adults, is expressed specifically in skeletal muscle, although inherited pathogenic mutations of its associated gene lead to cardiomyopathies [52]. Interestingly, a recent study also demonstrated that decreased MYLK2 expression occurred in the respiratory muscles of critically ill patients, in whom an increase in the proportion of myosin heads in the SRX and OFF state was also observed [53]. Further investigation into whether MYLK2 plays a functional role in the regulation of resting myosin conformation is essential, particularly given the high level of regulation of myosin by post-translational modifications. As a skeletal muscle-specific protein, MYLK2 could be a promising target for the modulation of resting myosin states in type 2 diabetes and related metabolic diseases in a way that would not impact the adult heart.

In our analysis of type II fibres, we did not observe changes to sarcomeric proteins. Instead, type II fibres demonstrated differential expressions of proteins associated with mitochondria. In particular, we observed significant changes to the expression of NADH:ubiquinone oxidoreductase subunit proteins, including NDUFS8, NDUFS5 and NDUFA10. These proteins have been previously demonstrated to be differentially expressed in the skeletal muscle of patients with type 2 diabetes [54]. As this is the first time (to our knowledge) that single-fibre proteomics has been used in the investigation of type 2 diabetes, this represents the first report that these proteins have specifically altered expression in type II fibres. Future experiments comprising fibre type-specific investigation of oxidative phosphorylation in individuals with and without type 2 diabetes would be of benefit as they would help to define whether mitochondrial dysfunction is fibre type-specific in type 2 diabetes.

The present study has several limitations. Our study only assessed myosin dynamics in male participants. Future studies evaluating sex-specific differences are warranted. This is particularly relevant when considering that it has already been demonstrated that oestradiol is able to bind to myosin molecules [55]. Furthermore, although the participants were similar between groups, they do fall into the older age range and overweight category. In addition, our specialised experimental methods, such as single-fibre analysis and x-ray diffraction readings, plus the scarce access to human skeletal muscle tissue from participants with similar characteristics, meant that the full complement of biophysical analyses could only be applied to a limited sample size (calculated through a prior power analysis).

### Conclusion

Our findings indicate that individuals with type 2 diabetes undergo a remodelling of their resting myosin SRX/OFF state in type I muscle fibres, leading to a lower ATP demand. Such (mal)adaptation may be related to aberrant glycation levels on myosin heavy chains, and may also be related to changes in the expression of sarcomeric proteins. Identification of these changes offers promising avenues for drug discovery, as they suggest that, if myosin biochemical and structural states were to be targeted pharmacologically and restored in type 2 diabetes, this would potentially help to increase the overall muscle/whole-body ATP consumption and thereby promote metabolic health.

## Supplementary Information

Below is the link to the electronic supplementary material.

Supplementary file1 (PDF 499 KB)

Supplementary file2 (XLSX 1217 KB)

## Data Availability

The raw MS data have been deposited to the ProteomeXchange Consortium via the PRIDE partner repository with the dataset identifier PXD053022 [56]. The rest of the data that support the findings of this study are available from the corresponding author upon reasonable request.

## Abbreviations

AGE: Advanced glycation end-products
CEL: *N*^ε^-(carboxyethyl)lysine
DRX: Disordered-relaxed state
Mant-ATP: 2′ (or 3′)-*O-*(*N-*methylanthraniloyl)-ATP
MG: Methylglyoxal
MG-H: Methylglyoxal-derived hydroimidazolones
MyHC: Myosin heavy chain
SRX: Super-relaxed state
