# Supplementary material for: Dysregulated skeletal muscle myosin super-relaxation and energetics in male participants with type 2 diabetes mellitus
Source: Diabetologia. 2025 Apr 28;68(8):1836–50. doi: 10.1007/s00125-025-06436-0 (PMC12245971; doi:10.1007/s00125-025-06436-0)

Supplementary information for

Dysregulated Skeletal Muscle Myosin Super-relaxation and Energetics in Males with Type 2 Diabetes Mellitus

This file includes:

ESM Table 1
ESM Figures 1 to 3

ESM Table 1. Quantification of Identified Proteins from Single Fibre Proteomics.
Detected protein abundance is listed for each individual muscle fibre analyzed. Significance levels are reported for each differential analysis performed. These are presented in the enclosed excel spreadsheet.

ESM Figure 1. Representative images of MYH7 positive staining in one permeabilized single fibre.

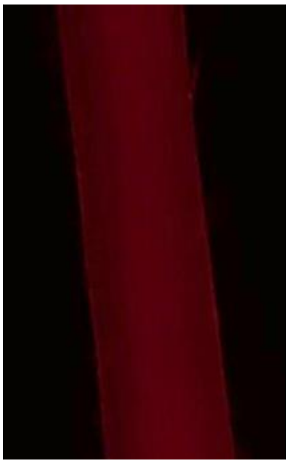

ESM Figure 2. Linear correlation between myosin dynamics and lean mass. a. Simple linear regression analysis between myosin DRX (P1) in MyHC type I muscle fibres and the lean mass (kg) of the subjects in both control and T2DM groups. **b.** Simple linear regression analysis between myosin DRX (P1) in MyHC type II muscle fibres and the lean mass (kg) of the subjects in both control and T2DM groups. Dotted lines demonstrate 95% confidence bands per group. *n* = 9-11.

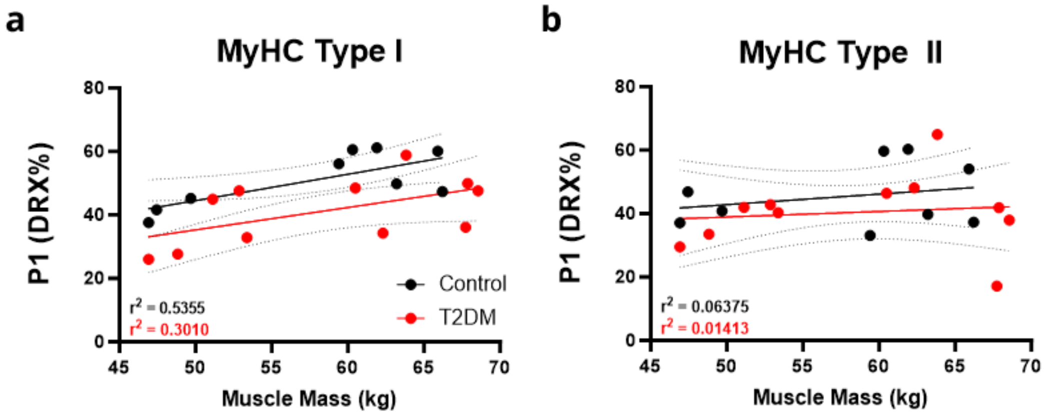

ESM Figure 3. Vseq characterization of AGE-modified peptide isoforms detected in MYH7. For each indicated peptide sequence the VseqExplorer characterization is demonstrated.

a

AK^{CEL}NALAHALQSAR

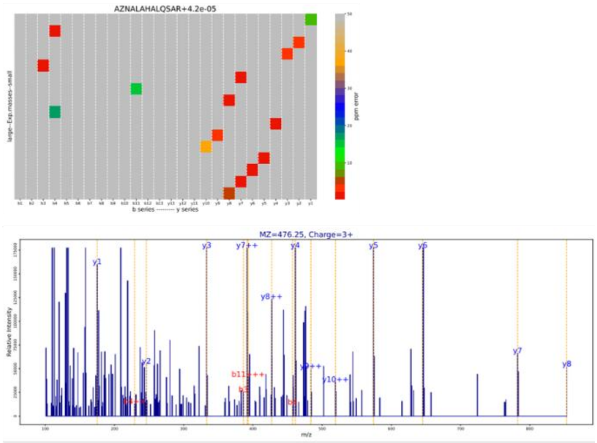

b

K^{CEL}MEGDLNEMEIQLSHANR

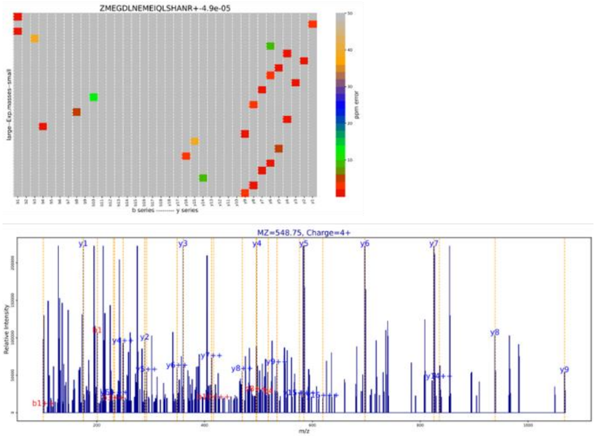

c

R$^{\text{MGH1}}$IK^{Carboxymethyl}ELTYQTEEDRK

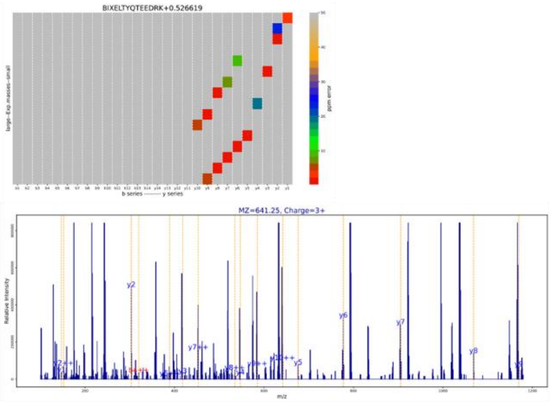

d

LAQR^{MGH1}LQEAEEAVEAVNAK

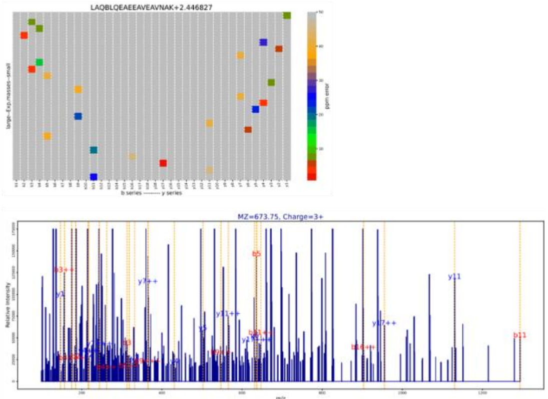

e

NHLR^{MGH1}VVDSLQTSLDAETR

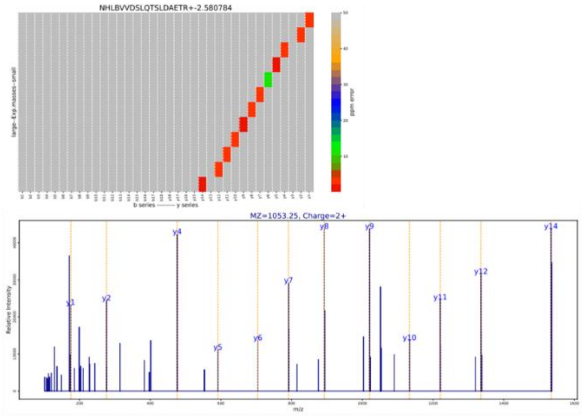

f

SR^{MGH1}K^{CEL}LAEQELIETSER

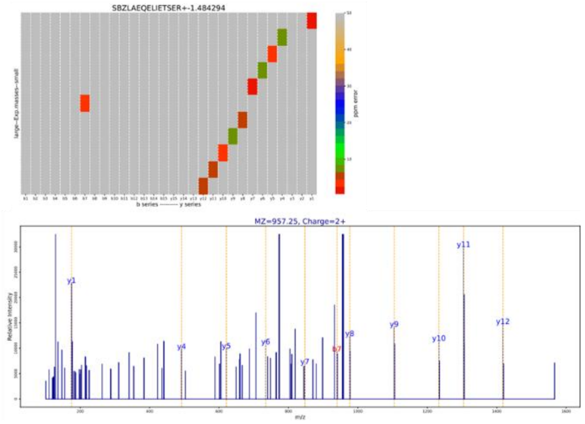

g

YR^{MGH1}ILNPAAIPEGQFIDSR

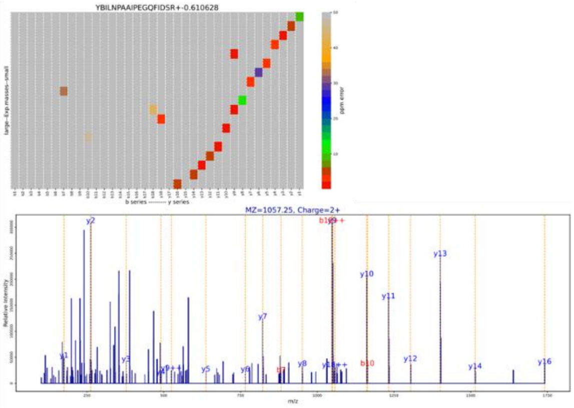

Supplement: Supplementary file 1 — Supplementary file1 (PDF 499 KB) [file 125_2025_6436_MOESM1_ESM.pdf]
